# The *Helicobacter pylori* single nucleotide polymorphisms SNPs associated with multiple therapy resistance in Colombia

**DOI:** 10.3389/fmicb.2023.1198325

**Published:** 2023-07-07

**Authors:** Kevin Guzman, Lidia Montenegro, Alvaro Pazos

**Affiliations:** ^1^Grupo Salud Pública, Centro de Estudios en Salud Universidad de Nariño (CESUN), Universidad de Nariño, Pasto, Colombia; ^2^Departamento de Biología, Universidad de Nariño, Pasto, Colombia

**Keywords:** resistance to antibiotics, *Helicobacter pylori*, evolution, gastric cancer, mutations

## Abstract

The eradication of *Helicobacter pylori* (*H. pylori*) using multiple therapies is used as a prevention strategy. However, its efficacy has been compromised by the emergence of single nucleotide polymorphisms in genes associated with *H. pylori's* resistance to multiple antibiotics. To estimate antibiotic resistance rates associated with mutations in *H. pylori* genes in the high-cancer-risk population in Colombia, we included 166 *H. pylori* whole genome sequences from a cohort of individuals with a high risk of gastric cancer. By using the reference strain ATCC 26695, we identified mutations in specific genes to evaluate resistance rates for different antibiotics: *23S* rRNA for clarithromycin, *16S* rRNA for tetracycline, *pbp1A* for amoxicillin, *gyrA* for levofloxacin, and *rdxA* for metronidazole. The phylogenomic analysis was conducted using the core genome consisting of 1,594 genes of *H. pylori*-ATCC 26695. Our findings revealed that the resistance rate of *H. pylori* to clarithromycin was 3.62%, primarily associated with mutations A2143G and A2142G in the *23S* rRNA gene. For tetracycline, the resistance rate was 7.23%, with mutations A926G, A926T, and A928C observed in the *16S* rRNA gene. Amoxicillin resistance was found in 25.9% of cases, with observed mutations in the *pbp1A* gene, including T556S, T593, R649K, R656P, and R656H. In the *gyrA* gene, mutations N87K, N87I, D91G, D91N, and D91Y were identified, resulting in a resistance rate of 12.04% to levofloxacin. The most common mutations in the *rdxA* gene associated with metronidazole resistance were a stop codon, and mutations at D59N and D59S, resulting in a resistance rate of 99.3%. The high resistance rate of *H. pylori* to metronidazole indicated that this drug should be excluded from the eradication therapy. However, the resistance rates for tetracycline and clarithromycin did not exceed the established resistance threshold in Colombia. The increased resistance rate of *H. pylori* to levofloxacin and amoxicillin may partially explain the observed therapeutic failures in Colombia. The phylogenomic tree showed that the *H. pylori* isolate belongs to its own lineage (hspColombia). These findings offer valuable insights to enhance the characterization of treatment protocols for the specific *H. pylori* lineage (hspColombia) at the local level.

## Introduction

*Helicobacter pylori* (*H. pylori*) is a microaerophilic, flagellated bacillus that is Gram-negative and pleomorphic and has been associated with pre-cancerous lesions and gastric cancer (GC) (Ishaq and Nunn, 2015; Baj et al., 2020). It has been estimated that half of the global population is infected with *H. pylori*, but <1% of them will progress to GC (Uemura et al., 2001). It has been described that the initial infection occurs during childhood from contaminated water or vegetables (Gomes and de Martinis, 2004; Goh et al., 2011).

Many virulence factors of *H. pylori* have been associated with cancer progression. The most frequent are the pathogenicity island *cag*PAI and the vacuolating cytotoxic VacA (Denic et al., 2020; Soluri et al., 2020). The pathogenicity island is a locus of 40 kb and has ~31 genes that encode for a type IV secretion system (Baj et al., 2020). The *cagA* gene encodes one of the oncoproteins that can be inserted inside the host's cells, leading to cell death (Soluri et al., 2020). The *vacA* gene is one of the most important virulence factors, and its high genetic diversity is associated with gastric wound development (Denic et al., 2020). The s1m1 alleles are the most pathogenic for the host compared to the s2m2 alleles (Atrisco et al., 2010; Román et al., 2017; Keikha et al., 2020).

Colombia has a high incidence of gastric cancer, and eradication of *H. pylori* from the gastric mucosa is an ideal treatment plan to revert gastric lesions, including peptic ulcers and chronic and atrophic gastritis (Matta et al., 2018). In Colombia, the first-line treatment for *H. pylori* infection consists of a standard triple therapy that includes a proton inhibitor, metronidazole, and amoxicillin. However, high levels of metronidazole resistance (81%) (Trespalacios et al., 2010) have forced us to change it to clarithromycin.

The therapeutic failure of first-line treatment in Colombia, associated with drug resistance, led to the use of tetracycline, clarithromycin, and levofloxacin as *H. pylori* rescue treatments (Camargo et al., 2014; Arévalo et al., 2019). Considering the increasing rates of bacterial antibiotic resistance, it is imperative to study mutations conferring drug resistance and its evolution to define novel evidence-based therapeutic schemes for *H. pylori* eradication in Colombia.

## Materials and methods

### Single mutations associated with antibiotic resistance in *Helicobacter pylori* isolates from colombia analysis

We used the published *H. pylori* genomes from Colombia. The studied sequences belong to the Cundiboyacense mountains (*n* = 131), Tolima region (*n* = 3), and Nariño region (*n* = 32) (https://pubmlst.org/). We reviewed the mutations associated with drug resistance in published studies on *LILACS, SciELO*, and *PubMed*. With that information, we built our database, which contained each mutation. Specifically, we checked for mutations on genes *pbp1A* for amoxicillin resistance, *23S* rRNA for clarithromycin, *16S* rRNA for tetracycline, *gyrA* for levofloxacin, and *rdxA* for metronidazole (Table 1). To determine the mutation on protein sequences, we analyzed the protein sequences that are encoded for the genes *pbp1A, 23S* rRNA, *16S* rRNA, *gyrA*, and *rdxA* of the reference strain 26695 (ATCC ID 700392) from the UniProt database (https://www.uniprot.org/) (Table 1). We extracted the protein sequences of 166 Colombian assemblies, annotated and aligned them with the Blast tool from the *H. pylori* collection genomes available in PubMLST (https://pubmlst.org/). The mutations were analyzed using the Uniprot UGENE v39.0 (Okonechnikov et al., 2012) software.

**Table 1:** Single nucleotide polymorphisms-SNPs in antibiotic multi-resistance genes of *Helicobacter pylori* in Colombia.

**Antibiotic**	**Gene**	**Studied SNPs**	**References**
Tetracycline	*16S* rRNA	A926T/A928C, A926G/G927T, A926G/A928C, A926G, and A939C	Trieber and Taylor (2002); Mannion et al. (2021)
Clarithromycin	*23S* rRNA	A2142G, A2142C, and A2143G	Taylor et al. (1997); Acosta et al. (2014); Roldán et al. (2019)
Amoxicillin	*pbp1A*	T556S, T593S, T593A, T593P, R649K, R656P, and R656H	Matteo et al. (2008); Tseng et al. (2009); Kwon et al. (2017)
Levofloxacin	*gyrA*	N87K, N87I, D91G, D91N, and D91Y	Tankovic et al. (2003); Trespalacios et al. (2016); Mannion et al. (2021)
Metronidazole	*rdxA*	D59N, R90K, H97T, H97Y, H97I, A118T, A118S, R131K, and I160F	Acosta et al. (2017)

### *Helicobacter pylori* phylogenomic analysis

Whole genome sequences were imported from the bacteria isolate genome sequence database (BIGSdb) (Jolley and Maiden, 2010). After that, a gene-by-gene alignment was done using the CDS sequences of the *H. pylori* reference strain 26695 (ATCC ID 700392) as a reference. The output matrix of the genome comparator obtained with BIGSdb, containing 1,694 genes from the *H. pylori* core genome, was used to make the phylogenomic tree using MEGA X (Kumar et al., 2018) with 1,000 bootstraps. The phylogenomic tree was viewed and edited in iTOL. v5 (Ivica and Peer, 2021).

## Results

**Figure 1:**
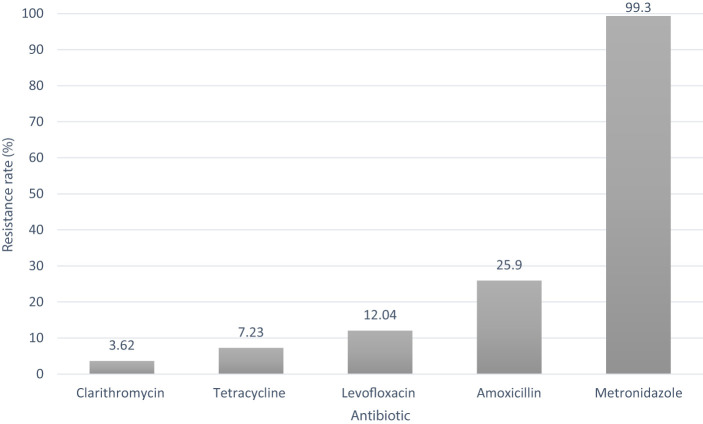
Antibiotic resistance rate of *Helicobacter pylori* isolates in Colombia.

Out of the 166 isolates, the proportion of genes associated with antibiotic resistance was 3.62% (6/166) for clarithromycin, 7.23% (12/166) for tetracycline, about 12.04% (20/166) for levofloxacin, 25.9% (43/166) for amoxicillin, and 99.3% (165/166) for metronidazole in Colombia (Figure 1). Mutations in the *23S* rRNA gene related to clarithromycin resistance were the most frequent. We found five isolates exhibiting the transition of adenine by guanine, A2142G, and only one isolate showed the transition of adenine by guanine in the 2,143 position (A2143G).

We observed 12 isolates carrying mutations in the gene *16S* rRNA related to tetracycline resistance. Nine isolates showed changes at the 926 position (either A926G or A926T), and three had the mutation at the position A928C. There were 43 mutations in the gene *pbp1A* associated with amoxicillin resistance. Among these mutations, 18 isolates exhibited transitions at position R649K, 13 isolates showed mutations at positions T593S, T593A, and T593P, eight isolates had mutations at positions R656P and R656H, and four isolates displayed the mutation at T556S. For *gyrA*, the gene related to levofloxacin resistance, 11 isolates showed the mutations D91G, D91N, and D91Y, and nine had the mutations N87K and N87I among the 20 evaluated isolates. Finally, regarding metronidazole resistance, we found 164 isolates with the mutation D59N, 78 isolates with the mutation R131K, 77 with the mutation R90K, 43 with the mutations H97T, H97Y, and H97I, 41 with the mutations A118T, A118S, and truncations in 34, 35, 50, and 51 positions in 1, 3, 10, and 1 isolates, respectively, in the gene *rdxA*.

We found 55 multidrug-resistant isolates. Five were resistant to clarithromycin and metronidazole; six to tetracycline and metronidazole; 32 to amoxicillin and metronidazole; four isolates were resistant to tetracycline, amoxicillin, and metronidazole; six were resistant to amoxicillin, levofloxacin, and metronidazole; and one was resistant to clarithromycin, levofloxacin, and metronidazole. Finally, we found one isolate with drug resistance-associated mutations to four antibiotics, including tetracycline, amoxicillin, levofloxacin, and metronidazole (Table 2).

**Table 2:** Multi-resistant *Helicobacter pylori* isolate to first- and second-line treatment in Colombia.

**Antibiotics-multi-resistance**	***n* multi-resistant isolates**	**Total**
Clarithromycin and metronidazole	5	
Tetracycline and Metronidazole	6	43
Amoxicillin and metronidazole	32	
Tetracycline, amoxicillin, and metronidazole	4	
Amoxicillin, levofloxacin, and metronidazole	6	11
Clarithromycin, levofloxacin, and metronidazole	1	
Tetracycline, amoxicillin, levofloxacin, and metronidazole	1	1
Total		55

First-line treatment: Clarithromycin, metronidazole, amoxicillin, and PPI; Second-line treatment: Tetracycline, levofloxacin, metronidazole, and PPI.

**Figure 2:**
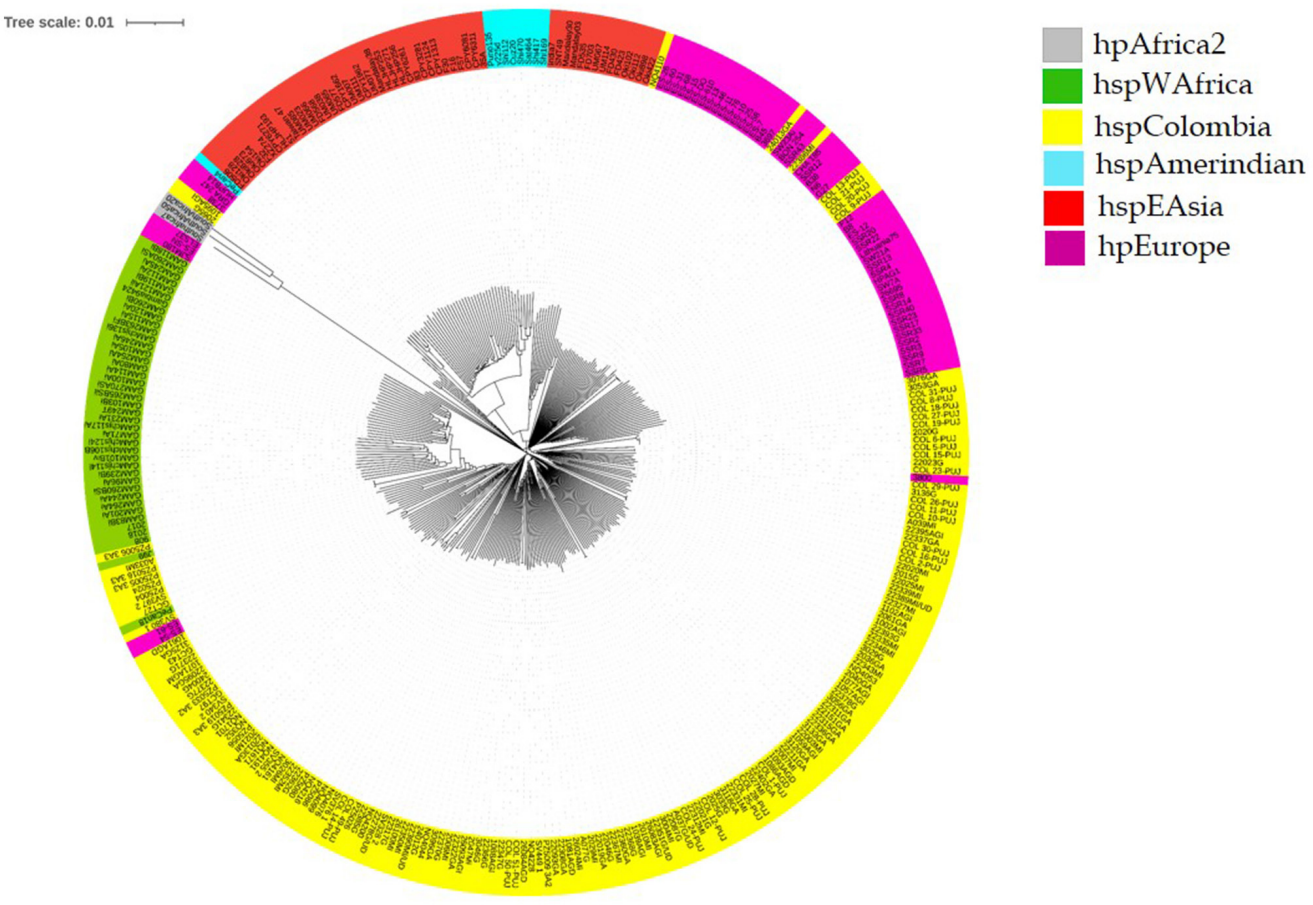
*Helicobacter pylori* phylogenomic tree of isolates from Colombia. Colombian isolates (hspColombia) are in yellow, violet for hpEurope strains, green for the West African continent (hspWAfrica), gray for South African continent strains (hpAfrica2), orange for East Asia isolates (hspEAsia), and cyan for Native American isolates (hspAmerindian).

In the core genome phylogenomic tree, we observed independent lineages of hpAfrica2, hspWAfrica, hpEAsia, hpEurope, hspAmerindian, and an independent clade of Colombian isolates (hspColombia) (Gutiérrez et al., 2017). We found that isolates clustered in the Native American clades had a close evolutionary relationship with Asian isolates (Kodaman et al., 2014). Moreover, in the cluster formed by Colombian isolates (hspColombia), we observed three hpEuropean and two hspWAfrican isolates. Finally, we found a group of seven isolates that were grouped with the de hpEurope linage, and only two isolates were grouped with hpAfrica2 (Figure 2).

## Discussion

In Colombia, there is a high prevalence of *H. pylori* infection (~80%) (Bravo et al., 2003) associated with gastric cancer patients, mostly in the Andean region. The genetic evolution of *H. pylori*, initially introduced in the Americas by the Europeans during colonization, due to adaptation to the Neotropical environmental features and the host genetics, led to the emergence of local strains (hspColombia) with resistant patterns that seem to be adjusted to the local therapy. In Colombia, it has been found that strains of African ancestry have high resistance to antibiotics despite their low virulence. However, strains from the Andean area show higher susceptibility than those from coastal Colombia (Figueroa et al., 2012). This finding may be linked to the strong African background found in the Colombian Pacific area, where *H. pylori* strains of African origin have coevolved with the human host, thereby allowing a higher contact with antibiotics used in treatments for multiple infections, including parasites and other bacteria, giving *H. pylori* the opportunity to develop resistance to the antibiotic. A different situation from Colombia has been described in the Andean mountains, where the coevolution of *H. pylori*-human seems to be disrupted. In these places, there is a high prevalence of new strains with less exposure to antibiotics and a lower resistance rate to antibiotics that are used for eradication, but also a higher incidence of gastric cancer (Figueroa et al., 2012; Matta et al., 2018; Mannion et al., 2021).

To eradicate the *H. pylori* infection, it is crucial to know the susceptibility and response to antibiotics to obtain the best treatment. In Colombia, the *H. pylori* treatment scheme is based on the combination of two antibiotics and a proton pump inhibitor such as omeprazole. Recently, the therapeutic efficacy of this triple therapy in Colombia has decreased due to the high resistance rate to antibiotics. For example, in Bogota, the proportion of resistance to metronidazole is ~97.3% (Yepes et al., 2008) (described by the E-test technique), almost the same as in our study, where we found 99.3% of resistance to this antibiotic, taking into account that most of the studied isolates in this study are from the Cundi-Boyacense mountains.

In this study, we found that D59N in *rdxA* was the most frequent mutation associated with resistance to metronidazole. This finding is in line with one study from the Cauca region that identified the same mutations with a frequency of 78.9% (Acosta et al., 2017). However, the resistance to metronidazole data could vary depending on the region of Colombia. For example, in several microbiological studies, the mean resistance ranges from 66 to 83% (Camargo et al., 2014).

Amoxicillin, a first-line antibiotic used for *H. pylori* eradication in Colombia, is at a high risk of failure due to the increased spread of resistant isolates ranging from 3.8 to 17% (Camargo et al., 2014; Atehortúa et al., 2020). We found mutations associated with resistance to amoxicillin as high as 25.9%. The highest frequency of amoxicillin resistance (20.5%) in Colombia was found in Tumaco City (Figueroa et al., 2012).

Clarithromycin is an antibiotic from the macrolide family that inhibits the protein synthesis of the *HPrrnB* region in the *23S* rRNA component. The frequency of mutations associated with resistance to this antibiotic was 3.62%, the lowest found compared to the other studied antibiotics. Colombia has been described as having a low resistance rate for this antibiotic, between 2.2 and 4% (Álvarez et al., 2009; Acosta et al., 2014). However, high resistance rates have been reported for this antibiotic, such as 15, 17.72, 18.8, 19.8, and 63.1% (Yepes et al., 2008; Henao et al., 2009; Trespalacios et al., 2010; Roldán et al., 2019), depending on the country regions. We observed a resistance rate of 7.23% for tetracycline harboring mutations associated with this antibiotic resistance. The most frequent changes found were in positions 926 (A926G and A926T) and A928C. However, in Colombia, the reported resistance rate to this antibiotic has been high, according to a previous study that reported a resistance rate of 85.7% (Yepes et al., 2008). Finally, levofloxacin is an antibiotic that has been used as a rescue therapy for unsuccessful first-line treatment. The main mutations in the gene *gyrA* have been associated with therapeutic failure to eradicate *H. pylori*. In Colombia, a resistance rate between 11.8 and 27.3% has been identified (Trespalacios et al., 2016). In this study, we found a 12.04% resistance rate, similar to previous studies.

The finding of strains with multi-resistance genotypes to two, three, and four antibiotics makes the hypothesis more correct about why the antibiotics fail in patients. Despite having assisted in controlling and treating the symptoms with the specialist, the symptoms and the *H. pylori* infection persist. In addition, we found multi-resistant isolates, especially one in the Nariño region, showing resistant genotypes to four antibiotics, which is why the World Health Organization (WHO) has declared *H. pylori* a priority in the search for new and effective antibiotics (World Health Organization, 2017).

Commercial kits have been developed based on real-time PCR to detect mutations in specific genes such as *23S* rRNA (A2142C, A2142G, and A2143G) and *gyrA* (N87K, D91G, D91N, and D91Y) to identify genotypic resistance from gastric tissue obtained by biopsies due to the time consuming and difficult *in vitro* culturing of *H. pylori* (Cambau et al., 2009; Scaletsky et al., 2011; Redondo et al., 2018), which could be applied as a new diagnosis and treatment strategy in Colombia.

However, one of our limitations is the possibility of other existing mutations in different genes at multiple sites that could affect the grade of resistance. For example, it is known that the gene *pbp1A* has an important role in amoxicillin resistance. This could be affected or regulated by other genes such as *pbp2, pbp3, hopC, hofH*, and the presence of the betalactamase TEM-1 in the bacteria (Domanovich-Asor et al., 2021).

Developing Next Generation Sequencing (NGS) technology will help find and suggest new *H. pylori* treatment models to prevent gastric pathogenesis through pathogen identification and genetic characterization like virulence genes, the evolutive lineage, and in the host, gastric cancer susceptibility genes (Malfertheiner et al., 2022). This study offers a preamble to antibiotic resistance mutation screening. However, it is necessary to conduct more studies that allow us to delve deeper into the *H. pylori* genome and the emergence of resistance (Malfertheiner et al., 2022).

The determination of antibiotic resistance-associated mutations by *H. pylori* in Colombia is of major importance in the treatment and eradication of bacterial infection. This study is the first to employ innovative diagnostic methods such as NGS, which offer significant advantages over traditional culture methods. These new methods enable faster and more accurate detection of mutations, which can positively impact the selection of effective treatments. Moreover, the successful application of NGS in Colombia can serve as a model for other countries and regions, accelerating global progress in diagnosing and treating antibiotic resistance in *H. pylori*.

## Conclusion

The phylogenomic tree showed that the *H. pylori* isolate belongs to an independent lineage, hspColombia, allowing the identification of a circulating strain in the Andean region from Colombia. We observed a high *H. pylori* resistance rate to metronidazole. It was suggested not to use this antibiotic in eradication therapies as a first-line treatment.

Clarithromycin and tetracycline are antibiotics that do not pass the established resistance rate threshold in the consensus for Colombia. Levofloxacin is an antibiotic whose resistance is increasing in Colombia. We suggest continuing surveillance of the resistance rates for this antibiotic.

## Data availability statement

The datasets presented in this study can be found in online repositories. The names of the repository/repositories and accession number(s) can be found below: https://pubmlst.org/organisms?title=Helicobacter+pylori.

## Ethics statement

Ethical review and approval was not required for the study on human participants in accordance with the local legislation and institutional requirements.

## Author contributions

KG and AP designed and coordinated the study, acquired and analyzed data, and interpreted the data. KG, LM, and AP wrote the manuscript and approved the final version of the article. All authors contributed to the article and approved the submitted version.
